# Determination of the Epitopes of Alpha-Glucosidase Anti-Drug Antibodies in Pompe Disease Patient Plasma Samples

**DOI:** 10.3390/antib14030064

**Published:** 2025-07-28

**Authors:** Evgeniy V. Petrotchenko, Andreas Hahn, Christoph H. Borchers

**Affiliations:** 1 Segal Cancer Proteomics Centre, Lady Davis Institute for Medical Research, Jewish General Hospital, McGill University, Montreal, QC H3T 1E2, Canada; evgeniy.petrotchenko@ladydavis.ca; 2 Department of Pediatric Neurology, University of Giessen, Feulgenstrasse 10-12, D-35389 Giessen, Germany; andreas.hahn@paediat.med.uni-giessen.de; 3 Gerald Bronfman Department of Oncology, McGill University, Montreal, QC H3T 1E2, Canada; 4 Division of Experimental Medicine, McGill University, Montreal, QC H3T 1E2, Canada; 5 Department of Pathology, McGill University, Montreal, QC H3T 1E2, Canada

**Keywords:** anti-drug antibodies, epitope mapping, enzyme replacement therapy, label-free quantitation, mass spectrometry

## Abstract

Pompe disease is a rare autosomal-recessive neuromuscular disorder caused by a deficiency of the lysosomal enzyme acid alpha-glucosidase (GAA), leading to the pathological accumulation of glycogen and impaired autophagy. Enzyme replacement therapy (ERT) with recombinant human alpha-glucosidase (rhGAA) has been available since 2006, but may lead to the formation of anti-drug antibodies (ADAs) against the recombinant human enzyme, which, in turn, may adversely affect the response to ERT. Knowledge of the antigenic determinants of rhGAA involved in interaction with ADAs may facilitate the development of strategies to attenuate the anti-drug immune response in patients. Here, we determined the rhGAA ADA epitopes in the plasma of Pompe disease patients using a series of affinity purifications combined with epitope extraction and label free quantitation LC-MS.

## 1. Introduction

The administration of protein-based drugs can lead to an immune response and the production of anti-drug antibodies (ADAs), which can diminish drug efficacy, affect drug pharmacokinetics, and cause other adverse effects [1,2]. Knowledge of the epitopes can be helpful for new approaches to predict, avoid, or even reverse such deleterious immune responses [3].

Pompe disease (also known as glycogen storage disease type II, acid maltase deficiency, or glycogenosis type II), is a rare autosomal recessive metabolic muscle disease [4]. A deficiency of acid alpha-glucosidase (GAA) results in lysosomal accumulation of glycogen (mainly in skeletal muscle), and severe deficiency leads to multisystem pathology and early death. GAA is a ~109 kDa glycoprotein with a complex glycosylation pattern [5]. The transfer of GAA to cells primarily occurs through mannose-6-phosphate (M6P) receptor-mediated endocytosis. GAA binds to M6P receptors on the target cell surface, the receptor-ligand complex is then internalized via clathrin-mediated endocytosis, transported to lysosomes, and activated for glycogen degradation. Alglucosidase alfa is a recombinant human GAA (rhGAA) produced in CHO cells that is used as enzyme replacement therapy (ERT) for Pompe disease since 2006. Alglucosidase alfa is a very complex mixture of glycoforms [5]. The development of high sustained levels of ADAs against rhGAA has been shown to substantially hamper drug uptake by target cells and reduce overall therapy efficiency, especially in children with complete GAA deficiency [6]. Knowing the anti-rhGAA epitopes may help to attenuate this immune response and guide the development of next-generation rhGAA drugs.

There are many technologies available for epitope mapping, including peptide arrays, electron microscopy, crystallography, and mutagenesis [7]. These approaches can be laborious, costly, and may require large protein amounts. Mass spectrometry-based techniques can be a viable alternative. Structural proteomics methods, such as limited proteolysis, surface modification, hydrogen-deuterium exchange (HDX), and crosslinking combined with modern mass spectrometry are capable of providing unique experimental data for protein structure determination [8]. Indeed, the protein drug–ADA complex is a particular type of protein-protein complex, for which structural proteomics methodology is well developed and has been broadly applied [9,10].

Epitope determination for ADA from patient samples, however, is challenging because of the polyclonal nature of the elicited ADAs and the minuscule amount of ADAs in the total immunoglobulin pool in plasma.

Very few studies of ADA epitope mapping from patient plasma samples have been reported thus far [11,12]. Schick et al. [12] used FPOP methodology to determine the epitopes of the recombinant bi-specific antibody for ADAs raised in goats and monkeys. Grauslund et al. [13] used HDX for the determination of polyclonal ADA epitopes of recombinant protein used for vaccination. One of the earliest and most well-developed structural proteomics approaches for epitope mapping is limited proteolysis combined with mass spectrometry (LiP-MS) [14]. Two variations of the method have been used which can potentially determine both linear and conformational epitopes. In epitope extraction, the protein antigen is digested with proteolytic enzymes and the peptides which bind to the antibody are considered to contain the epitope. In epitope excision, the protein antigen-antibody complex is proteolytically digested while the antigen is still bound to the antibody, and the portion of the antigen still bound to the antibody is considered to contain the epitope [15].

Here, we performed proof-of-principle epitope mapping of anti-rhGAA ADAs from a minimal amount of plasma from a Pompe-disease patient using a novel variant of epitope extraction with affinity purification and LC-MS with label-free quantitation.

## 2. Experimental Procedures

*Samples and materials.* Leftover anonymized patient plasma material collected at the University of Giessen hospital was used for the study. Approval from the Institutional Review Board (IRB) Ethics Committee of the Jewish General Hospital was in place as part of the quality assurance program (Quality Program, 28 May 2020). The research was performed in accordance with the Declaration of Helsinki (European Council 2001, US Code of Federal Regulations, ICH 1997).

*Epitope extraction.* 50 µL of plasma samples from an anonymized Pompe-disease patient and healthy control plasma (K2 plasma, BioIVT) were subjected to affinity purification of the anti-rhGAA ADAs using rhGAA protein covalently immobilized on agarose beads. Affinity beads were prepared with 100 µL of a 5 mg/mL solution of Myozyme (alglucosidase alfa). 10 µL of 0.1 M sodium bicarbonate was added and incubated with 10 mg of NHS-Sepharose (Sigma, St. Louis, MO, USA) overnight at room temperature (23 °C) with end-over-end mixing. The beads were washed three times with 1 mL of 50 mM of ammonium bicarbonate, incubated for 1 h at room temperature to block any remaining active NHS-groups, and washed with 1 mL of PBS. 20 µL of 50% bead slurry were combined with 50 µL of control and patient plasma and incubated for 1 h at room temperature with end-over-end mixing. Beads were washed three times with 10 µL PBS and the bound ADAs were competitively eluted for 1 h at room temperature with 50 µL of peptides from a tryptic digest of the rhGAA protein that had been quenched with PMSF. The digest was prepared by incubating 100 µL of a 5 mg/mL solution of Myozyme with 10 µL of a 10 mg/mL solution of trypsin (Worthington) and 20 µL of 0.1 M sodium bicarbonate overnight at 37 °C. Residual trypsin activity was inhibited by adding 2 µL of 0.1 M PMSF. The eluted antibody-peptide complexes were captured on 20 µL of Protein A/G magnetic beads (Thermo, Waltham, MA, USA) incubated for 1 h at room temperature and washed with PBS. The beads were washed four times with 20 µL PBS, and once with 200 µL of water, and the bound peptides were eluted with 100 µL of an aqueous 0.1% formic acid solution.

*LC-MS analysis.* Eluted peptides from the patient and control samples were loaded on Evotips (Evosep, Odense, Denmark) and analyzed by LC-MS using Orbitrap QE+ coupled to an Evosep One UPLC system. Full MS scans were acquired from *m/z* 350 to *m/z* 1500 at a resolution of 70,000, with an automatic gain control (AGC) target value of 1 × 10^6^, and a maximum injection time of 50 ms. The 15 most intense precursor ions (charge states +2 to +8) were isolated with a window of 1.2 Da and fragmented using a normalized higher-energy collisional dissociation (HCD) energy of 28, and a dynamic exclusion of 40 s. The MS/MS spectra were acquired at a resolution of 17,500 using an AGC target value of 2 × 10^4^ and a maximum injection time of 64 ms. Differential label free quantitation data analysis using the Quant MS1 module of the FragPipe v19.1 [16] was used to identify ADA-bound rhGAA peptides.

*Experimental Design and Statistical Rationale.* A single control sample and a single patient sample were analyzed for epitope determination using epitope extraction of ADAs from plasma as a proof-of-principle approach. The intensity-ratio cutoff thresholds for discriminating peptides specifically bound to ADAs rhGAA were chosen based on the observed bimodal distribution of the log-transformed ratios.

## 3. Results and Discussion

**Figure 1:**
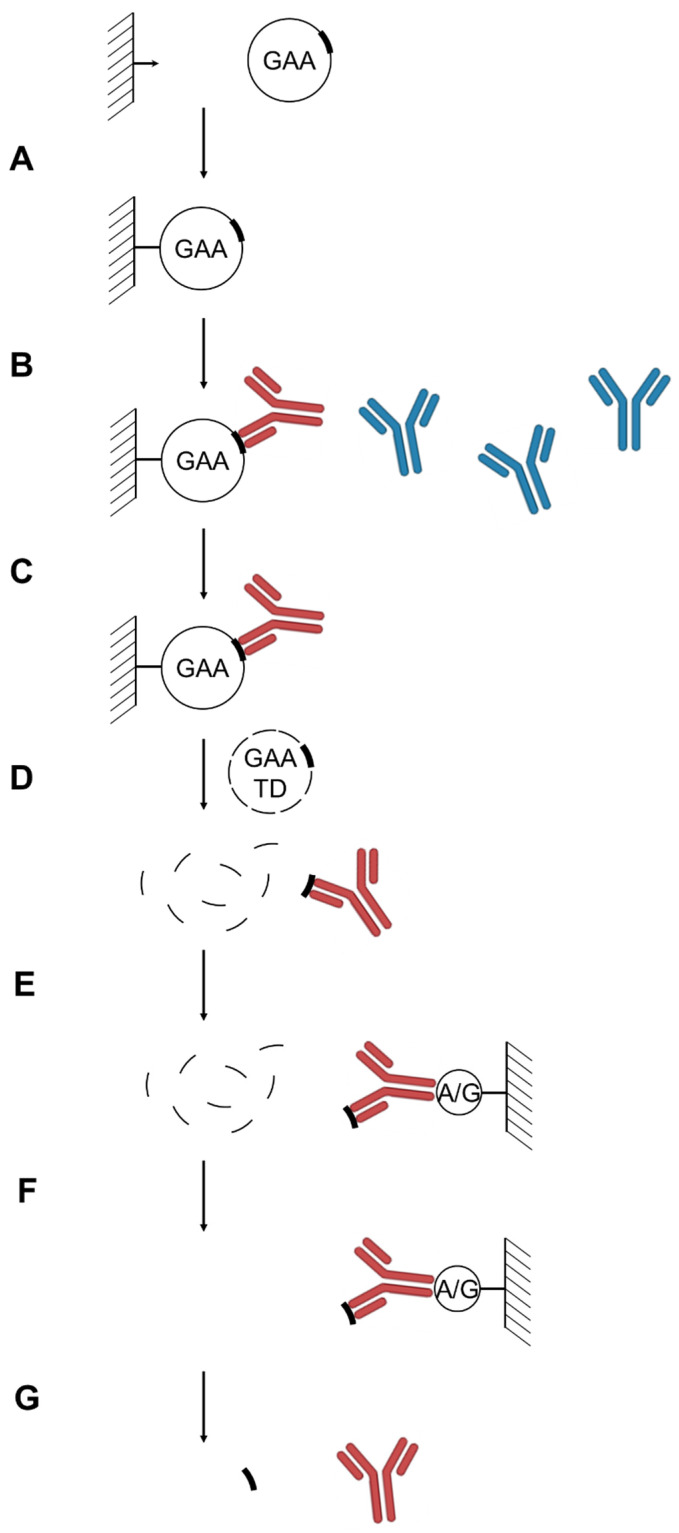
Analytical scheme of the anti-rhGAA anti-drug antibodies epitopes determination. (**A**) rhGAA immobilization. (**B**) anti-rhGAA ADA pull-down from patient plasma samples. (**C**) Wash. (**D**) Competitive elution of anti-rhGAA ADAs with rhGAA trypsin digest. (**E**) anti-rhGAA ADA pull-down by Protein A/G. (**F**). Wash. (**G**). Low pH elution of ADA-bound rhGAA epitope-containing tryptic peptides for subsequent identification by LC-MS. Anti-rhGAA ADAs are shown in red, non-specific immunoglobulins are shown in blue; epitope-containing and non-epitope-containing rhGAA peptides are shown as thick and thin lines, respectively.

*Determination of the alglucosidase alfa ADA epitopes.* Epitope extraction analysis was used for the identification of the rhGAA epitopes for the anti-rhGAA ADAs isolated from the Pompe disease patient plasma samples. The antibodies were enriched on immobilized rhGAA beads and were competitively eluted with a rhGAA tryptic digest peptide mixture. The antibody-peptide complexes were further purified on Protein A/G beads, the antibody-bound peptides were then eluted with a low-pH solution (here, aqueous 0.1% formic acid solution) and analyzed by LC-MS (Figure 1). Due to the small amount of the sample and the amount of ADA in the sample, distinguishing specifically bound from non-specifically bound peptides is of critical importance. To address this, we used a comparison with the untreated plasma as a control. All of the purifications steps were done in parallel using the same procedures and materials for the control and patient samples. Differential label free quantitation analysis of the patient and healthy control plasma samples was then used to distinguish between specifically bound and non-specifically bound peptides (which may appear in both samples). We used FragPipe MS1 quantitation module to compare abundancies of the rhGAA peptides between samples. A fold change of 4.0 was used as the cutoff for peptide intensity ratios (Table 1). We did not detect any glycosylated peptides differentially present in the samples, even though the major glycosylated peptide forms [5] were included in the search.

The proposed workflow requires only a minimal amount of patient plasma, uses straightforward preparation of the affinity sorbent with immobilized protein drug (which is usually available in abundance), and standard reagents. Epitope-containing peptides can be identified using label-free quantitation-enabled LC-MS, by comparing the patient and control plasma samples which are processed in parallel. The approach described here can potentially be applied to the determination of the epitopes of other protein-drug ADAs.

**Figure 2:**
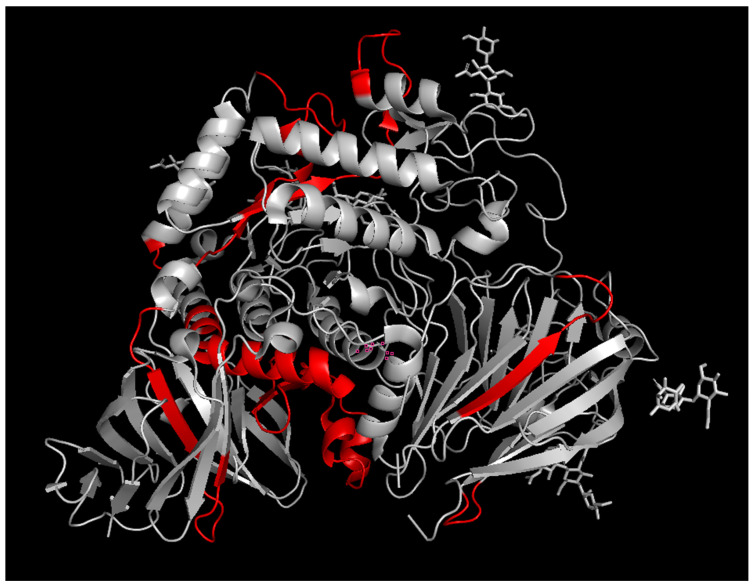
The epitopes of rhGAA for anti-drug antibodies determined by epitope extraction analysis. Epitope-containing peptide sequences are highlighted in red.

*Alglucosidase alfa ADA epitopes.* The sequences of the peptides that were preferentially present in the patient samples were mapped onto three-dimensional structure of rhGAA (PDB 5NN8) (Figure 2). The epitopes found were mainly located in the loops on the surface of the protein. The epitopes determined were mainly clustered at the surfaces of the central part of the catalytic GH31 domain, at the interface between GH31 and C-terminal beta-sheet domain, and at the N-terminal beta-sheet domain. The central catalytic domain epitope cluster is located in the vicinity of the substrate binding site and may indicate potential inhibition of the enzyme activity by ADA, if still bound inside the target cells. To exert its action, rhGAA has to be internalized into the cells via the mannose-6-phosphate receptor, which binds to the corresponding terminal phosphorylated mannose residues of GAA glycans. The phosphorylated mannose residues in alglucosidase alfa were determined to be located mainly at the N140 and N470 glycosylation sites [5]. Thus, the binding of anti-rhGAA ADA to the catalytic and/or N-terminal beta-sheet domain epitope clusters may attenuate the receptor binding. The fact that we did not detect any glycosylated peptides bound to ADA rhGAA could be due to the lack of antibodies directed to the carbohydrate moieties of the glycoprotein, weak glycan-antibody interactions, or could be due to the complex glycosylation pattern of alglucosidase alfa leading to the dilution of the signal and difficulties in the identification of the glycopeptides. The first hypothesis is in agreement with reports suggesting that, in some cases, anti-rhGAA ADAs do not inhibit receptor-mediated uptake of the drug [17], inferring that the ADAs are not directed toward M6P residues. In future studies, it would be interesting to compare ADA titres and the ADA epitope distributions for the next generation of rhGAA drugs, such as avalglucosidase alfa or cipaglucosidase, with increased content of M6P. If our observations hold true for all glycans, the increased glycosylation of protein drugs may serve as a general strategy for avoiding anti-drug host immune responses by preventing antibodies from binding to the protein drug surface or interfering with the presentation of the protein drug antigens to immune cells. 

## 4. Conclusions

We have developed an analytical workflow for the determination of the epitopes of protein drug ADAs from a minimal amount of patient plasma and used it for the determination of the anti-rhGAA ADA epitopes from Pompe-disease patient plasma samples. Our workflow includes a pulldown of ADAs with an immobilized drug, elution of the bound ADAs with a tryptic digest of the drug, pulldown of peptide-ADAs complexes, and identification of the peptides bound to ADAs. The developed approach is generally applicable for the characterization of ADAs in other protein-based therapies. Identified anti-rhGAA ADA epitopes may facilitate development of the next generation of rhGAA drugs.

## Figures and Tables

**Table 1:** rhGAA peptides, containing epitopes for anti-drug antibodies.

Peptide Sequence	Start	End
LHFTIKDPANR	179	189
QLDGR	225	229
AHFPLDVQWNDLDYMDSRRDFTFNK	394	418
DFPAMVQELHQGGRRYMMIVDPAISSSGPAGSYR	438	456
ARGTRPFVISR	590	600
ALTLRYALLPHLYTLFHQAHVAGETVAR	698	725
YALLPHLYTLFHQAHVAGETVAR	703	725
PLFLEFPKDSSTWTVDHQLLWGEALLITPVLQAGK	726	760
QQPMALAVALTKGGEAR	838	854

## Data Availability

LC-MS data has been uploaded to PRIDE repository (PXD065994), all other data are available upon request.

## Abbreviations

GAA, acid alpha-glucosidase; ERT, enzyme replacement therapy; rhGAA, recombinant human alpha-glucosidase, alglucosidase alfa; ADA, anti-drug antibody; M6P, mannose-6-phosphate; FPOP, fast photochemical oxidation of proteins; HDX, hydrogen deuterium exchange; LiP-MS, limited proteolysis combined with mass spectrometry; LC-MS, liquid chromatography combined with mass spectrometry.
